# Leveraging Social Media for Health Education: Dissemination of Crohn’s Perianal Fistula Educational Videos via Facebook and Instagram

**DOI:** 10.1093/crocol/otaf060

**Published:** 2025-10-21

**Authors:** Jan M Ballesteros, Muskaan Mehra, So Yung Choi, Zoe Krut, Mike Simpson, Yixin Yang, Carine Khalil, Yee Hui Yeo, Brennan M R Spiegel, Christopher V Almario

**Affiliations:** Cedars-Sinai Center for Outcomes Research and Education (CS-CORE), Los Angeles, CA, United States; Department of Medicine, Cedars-Sinai Medical Center, Los Angeles, CA, United States; Cedars-Sinai Center for Outcomes Research and Education (CS-CORE), Los Angeles, CA, United States; Department of Medicine, Cedars-Sinai Medical Center, Los Angeles, CA, United States; Cedars-Sinai Center for Outcomes Research and Education (CS-CORE), Los Angeles, CA, United States; Department of Medicine, Cedars-Sinai Medical Center, Los Angeles, CA, United States; Cedars-Sinai Center for Outcomes Research and Education (CS-CORE), Los Angeles, CA, United States; Department of Medicine, Cedars-Sinai Medical Center, Los Angeles, CA, United States; Variant Media, Inc., Temecula, CA, United States; Cedars-Sinai Center for Outcomes Research and Education (CS-CORE), Los Angeles, CA, United States; Department of Medicine, Cedars-Sinai Medical Center, Los Angeles, CA, United States; Cedars-Sinai Center for Outcomes Research and Education (CS-CORE), Los Angeles, CA, United States; Department of Medicine, Cedars-Sinai Medical Center, Los Angeles, CA, United States; Cedars-Sinai Center for Outcomes Research and Education (CS-CORE), Los Angeles, CA, United States; Department of Medicine, Cedars-Sinai Medical Center, Los Angeles, CA, United States; Karsh Division of Gastroenterology and Hepatology, Cedars-Sinai Medical Center, Los Angeles, CA, United States; Cedars-Sinai Center for Outcomes Research and Education (CS-CORE), Los Angeles, CA, United States; Department of Medicine, Cedars-Sinai Medical Center, Los Angeles, CA, United States; Karsh Division of Gastroenterology and Hepatology, Cedars-Sinai Medical Center, Los Angeles, CA, United States; Division of Health Services Research, Cedars-Sinai Medical Center, Los Angeles, CA, United States; Cedars-Sinai Center for Outcomes Research and Education (CS-CORE), Los Angeles, CA, United States; Department of Medicine, Cedars-Sinai Medical Center, Los Angeles, CA, United States; Karsh Division of Gastroenterology and Hepatology, Cedars-Sinai Medical Center, Los Angeles, CA, United States; Division of Health Services Research, Cedars-Sinai Medical Center, Los Angeles, CA, United States

**Keywords:** Crohn’s perianal fistula, health education, social media, patient engagement

## Abstract

**Background:**

Crohn’s perianal fistula (CPF) is a debilitating disease that significantly affects patients’ quality of life. Prior research revealed substantial knowledge gaps among individuals with CPF in accessing evidence-based educational resources. We evaluated the reach and engagement of two validated CPF educational videos and a companion website (HealMyFistula.org) that was disseminated through Facebook and Instagram.

**Methods:**

A six-month social media campaign was conducted to promote the CPF educational videos and website using targeted advertising strategies to maximize reach. Primary outcomes were the proportion of users who viewed at least 50% of a video and the outbound click rate to the website. Results were stratified by age, sex, and social media platform.

**Results:**

The CPF Overview video reached 3.1 million users and had a ≥ 50% view rate of 3.31% (*n* = 103 463), while the CPF Treatment video reached 1.8 million users with a 1.40% view rate (*n* = 25 606). Older age groups showed significantly higher engagement, while females demonstrated higher view and click rates to HealMyFistula.org than males (*P* < .05). Facebook outperformed Instagram for the CPF Overview video, while Instagram slightly outperformed Facebook for the CPF Treatment video.

**Conclusions:**

Targeted social media campaigns can be effective in disseminating educational content. Engagement varied by video type, age, sex, and platform, showing the importance of demographically informed dissemination strategies. These findings suggest that health educational campaigns may benefit from tailoring content and dissemination strategies based on demographic engagement patterns.

## Graphical abstract

**Graphical Abstract:**
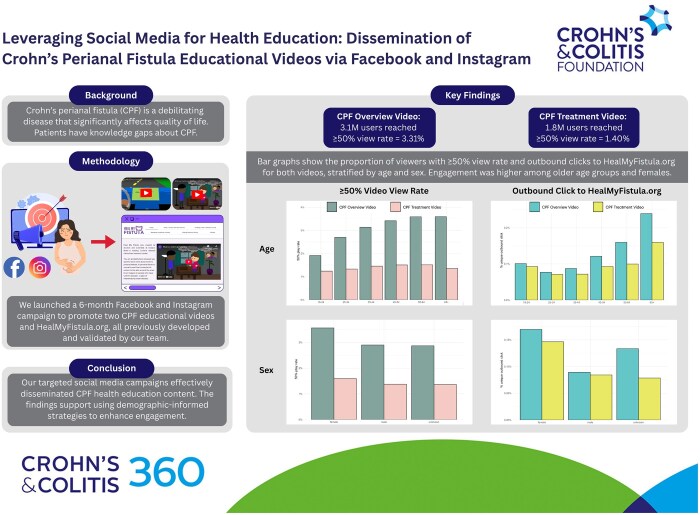

## Introduction

Crohn’s disease is a chronic inflammatory disease characterized by transmural inflammation that disrupts the mucosal integrity of the intestine and anal canal, leading to complications such as fistulae.^1^ The prevalence of Crohn’s perianal fistula (CPF) is estimated at 11%-21% within 1 year of Crohn’s disease diagnosis, and between 21%-54% for patients within 20 years of their diagnosis.^2^^,^^3^

Although CPF is common in Crohn’s disease and significantly impacts biopsychosocial wellbeing,^4^ its management remains challenging.^3^ Moreover, our research group found evidence of knowledge gaps among patients with CPF through our analysis of posts made on social media and e-health forums.^5^ Among the 119,986 analyzed posts, 66% were made by individuals who were seeking and sharing information regarding CPF, including on how it is diagnosed, common symptoms, and treatment options.^5^

**Figure 1.**
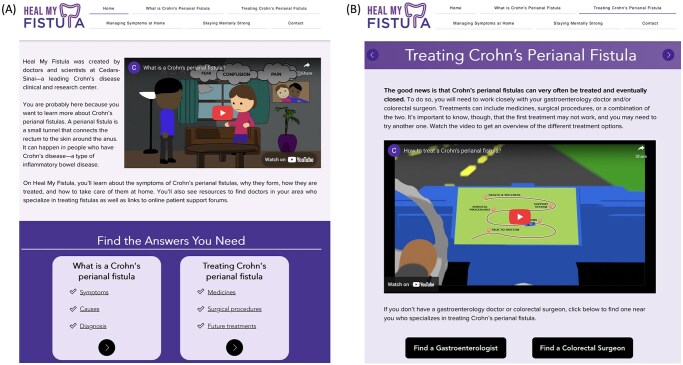
Screenshots of the Heal My Fistula (HealMyFistula.org) home page (Panel A) and Treating CPF page (Panel B). The website was iteratively developed through a user-centered design in partnership with patients with CPF, gastroenterologists, and colorectal surgeons.

Given the limited availability of authoritative CPF resources,^6^ our research group created 2 educational videos optimized for social media dissemination and a website called Heal My Fistula (HealMyFistula.org; Figure 1). One video provided an overview of CPF while the other described the various treatment options. The educational assets were developed using a user-centered design approach in collaboration with gastroenterologists, colorectal surgeons, and patients with CPF.^7^ In a separate mixed-methods study, we showed that the videos significantly improved patients’ CPF knowledge and engagement.^7^ As part of this current study, we aimed to evaluate the reach of the videos and website through a 6-month social media marketing campaign, leveraging targeted advertising on Facebook and Instagram to widely promote and disseminate the videos to people with CPF and those who know someone with CPF.

## Materials and Methods

Two validated videos^7^ were disseminated on Facebook and Instagram within the US between February 27, 2024, and September 16, 2024, using the Facebook Ads Manager. One video provided an overview of CPF (“What is CPF?”; herein after referred to as the CPF Overview video) while the other outlined treatment options (“How to treat CPF?”; ie, CPF Treatment video).

Both videos were about 2.5 minutes long (CPF Overview video—2:21 minutes; CPF Treatment video—2:33 minutes) and were accompanied by descriptive text summarizing the content and directing viewers to the Heal My Fistula website:

1. **CPF Overview video description:** 
*Crohn’s perianal fistulas can cause confusion and embarrassment for those with Crohn’s disease. When you are not feeling well, the journey towards healing may seem daunting. Watch the video for insights into how fistulas form, their symptoms, and the essential first steps on the path to recovery.*
*Want to learn more? Visit*  *Heal My Fistula**, a resource crafted by doctors and scientists at Cedars-Sinai—a leading Crohn’s disease clinical and research center.*
2. **CPF Treatment video description:** 
*Living with Crohn’s perianal fistula can be stressful and make you feel like you are driving on a windy, bumpy road. But there are checkpoints you can make on your road to recovery. Watch the video to explore the treatments that can close the fistula such as medications and surgical procedures.*
*Want to learn more? Visit* *Heal My Fistula* *, a resource crafted by doctors and scientists at Cedars-Sinai—a leading Crohn’s disease clinical and research center.*

To identify relevant viewers (ie, people with CPF or those who know someone with CPF) and maximize video engagement on Facebook and Instagram, we used the following approaches:

1. **Interest-Based Audiences:** Users who demonstrated interest in a topic by engaging with related content, posting about it, or visiting relevant websites. Facebook and Instagram assigned unique interest categories to users based on their activities, and preset audiences were created accordingly.
2. **Retargeting:** Users who previously engaged with the videos, website, or related posts were placed on a retargeting list for further exposure to campaign content.
3. **Behavioral (Lookalike) Audiences:** The platform identified larger groups of users exhibiting similar online behaviors to a known engaged audience, such as individuals who watched ≥50% of a video. This approach expanded reach by targeting users with a high likelihood of interest in CPF-related content.

The primary outcomes were the percentage of targeted users who viewed ≥50% of a video and the outbound click rate to Heal My Fistula. Outcomes were also stratified by age, sex, and social media platform.

## Results

### ≥50% video view rate

The videos appeared in the feeds for >5 million Facebook and Instagram users (CPF Overview video—3.1M; CPF Treatment video—1.8M). For the CPF Overview video, 103,463 (3.31%) people watched ≥50% of the video, compared to 25,606 (1.40%) for the CPF Treatment video (*P* < .001).

Increasing age was associated with higher ≥50% view rates for both videos (*P* < .001; Table 1). For example, for the CPF Overview video, only 1.91% of people aged 18-24 years watched at least half the video versus 3.59% of those aged 55-64 and ≥65 years. As for sex, females were more likely to watch both videos when compared to males and those whose sex was unknown (both videos with *P* < .001; Table 1). For instance, 3.57% of females watched ≥50% of the CPF Overview video versus 2.90% and 2.87% of males and those with unknown sex, respectively.

**Table 1.** ≥50% view and outbound click rates for the CPF Overview and CPF Treatment videos, stratified by age, sex, and social media platform.

≥50% view rate	CPF Overview video % (95% CI)	*P*-value	CPF Treatment video % (95% CI)	*P*-value
**Age: 18-24 y**	1.91 (1.83, 2.00)	<.001	1.24 (1.20, 1.29)	<.001
**25-34y**	2.70 (2.64, 2.76)		1.33 (1.30, 1.36)	
**35-44 y**	3.14 (3.09, 3.18)		1.46 (1.42, 1.49)	
**45-54 y**	3.41 (3.37, 3.46)		1.51 (1.46, 1.55)	
**55-64 y**	3.59 (3.55, 3.63)		1.51 (1.46, 1.56)	
**≥65 y**	3.59 (3.54, 3.64)		1.35 (1.31, 1.41)	
**Sex: Female**	3.57 (3.54, 3.59)	<.001	1.59 (1.53, 1.65)	<.001
**Male**	2.90 (2.87, 2.93)		1.38 (1.36, 1.39)	
**Unknown**	2.87 (2.72, 3.02)		1.36 (1.21, 1.53)	
**Platform: Facebook**	3.35 (3.32, 3.37)	<.001	1.37 (1.35, 1.39)	<.001
**Instagram**	2.78 (2.70, 2.85)		1.49 (1.45, 1.52)	

Outbound click rate	CPF Overview video % (95% CI)	*P*-value	CPF Treatment video % (95% CI)	*P*-value
**Age: 18-24 y**	0.10 (0.08, 0.12)	<.001	0.09 (0.08, 0.11)	<.001
**25-34y**	0.08 (0.07, 0.09)		0.07 (0.06, 0.08)	
**35-44 y**	0.09 (0.08, 0.09)		0.07 (0.06, 0.08)	
**45-54 y**	0.12 (0.11, 0.13)		0.09 (0.08, 0.11)	
**55-64 y**	0.16 (0.15, 0.17)		0.10 (0.09, 0.11)	
**≥65 y**	0.24 (0.23, 0.25)		0.16 (0.14, 0.18)	
**Sex: Female**	0.17 (0.16, 0.18)	<.001	0.15 (0.13, 0.16)	<.001
**Male**	0.09 (0.08, 0.09)		0.08 (0.08, 0.09)	
**Unknown**	0.13 (0.10, 0.17)		0.08 (0.05, 0.13)	
**Platform: Facebook**	0.14 (0.14, 0.14)	.24	0.09 (0.08, 0.09)	.17
**Instagram**	0.13 (0.11, 0.15)		0.10 (0.09, 0.11)	

CI, confidence interval; CPF, Crohn’s perianal fistula.

When considering social media platform, the CPF Overview video had a higher ≥50% view rate on Facebook (3.35%) when compared to Instagram (2.78%, *P* < .001; Table 1). On the other hand, the CPF Treatment video had a slightly higher view rate on Instagram (1.49%) over Facebook (1.37%, *P* < .001; Table 1).

### Outbound click rate to Heal My Fistula website

The CPF Overview video had a higher outbound click rate of 0.14% to the Heal My Fistula website compared to the CPF Treatment video with 0.09% (*P* < .001). In general, for both videos, increasing age was associated with higher outbound clicks (*P* < .001; Table 1). Females also had higher outbound click rates for both videos (*P* < .001; Table 1). For example, 0.15% of females who were shown the CPF Treatment video visited Heal My Fistula when compared to 0.08% of males and 0.08% of those with unknown sex (*P* < .001). Similarly, for the CPF Overview video, the outbound click rate was highest among females at 0.17% compared to males at 0.09% and those with unknown sex at 0.13% (*P* < .001). As for social media platforms, no difference in outbound click rates was seen between Facebook and Instagram for either video (Table 1).

## Discussion

Through our social media campaign, we broadly disseminated the validated CPF videos and Heal My Fistula website^7^ across Facebook and Instagram. As a result, over 125,000 users watched at least half of a video, and more than 6,000 visited the website.

Our study has several notable findings. First, we observed a higher ≥50% view rate for the CPF Overview video—which explained CPF development, associated symptoms, and treatment options—compared to the CPF Treatment video, which focused on specific medications and surgical procedures for CPF management. Both educational videos were similar in length (approximately 2.5 minutes), minimizing the likelihood that differences in view rates were driven by video duration. Instead, observed differences likely reflect variation in video content or audience interest. This suggests that people on social media are more interested in broadly understanding CPF itself rather than seeking detailed information about its treatment. For investigators aiming to maximize engagement with educational content on social media, prioritizing broad, introductory resources over highly specific, in-depth materials may be a more effective strategy.

Second, we observed differential ≥50% view rates and outbound click rates for both videos across age and sex. Overall engagement increased with age, peaking among individuals aged 55 years and older. The reasons for this trend are unclear but may be due to older individuals with Crohn’s disease being more likely to have experienced a CPF at some point in their disease course.^8^ Among those without Crohn’s disease, this pattern could reflect a higher likelihood of knowing someone with CPF, greater trust in online educational content, or a higher propensity to seek health-related information. Additionally, females demonstrated higher engagement with both videos compared to males and those of unknown sex. This may be attributed to known differences in health information-seeking behavior, as females are generally more likely to research health-related topics.^9^ To ensure online educational content effectively meets audience needs, investigators may consider oversampling women and older individuals during the content co-development process.

The success of our campaign highlights the power of social media as a scalable and targeted tool for health education and outreach. Platforms like Facebook and Instagram offer robust tools for demographic targeting, behavioral tracking, and real-time engagement analysis, which can be leveraged by researchers to identify and reach niche populations efficiently. This approach is particularly useful for conditions with low prevalence such as CPF, and where traditional recruitment and outreach methods may be inefficient or costly. Compared to in-person education or printed informational campaigns, social media offers a cost-effective strategy for reaching large and diverse audiences quickly, while also providing immediate feedback through engagement metrics. Future studies could apply similar designs not only to assess educational reach but also to test different messaging strategies or recruitment methods for digital or traditional health interventions—including on other widely used platforms such as TikTok, Snapchat, and Reddit, among others.

This study has limitations. First, both view rates and outbound click rates were relatively low overall, which is expected given that Crohn’s disease and CPF are not highly prevalent.^10^ Second, while we showed differences in engagement by age and gender with respect to ≥50% view and outbound click rates, we did not fully characterize how users interacted with the materials. Future studies may consider assessing additional social media metrics (e.g., likes, comments, shares) and website metrics (e.g., time spent on page, bounce rate) to provide a more comprehensive understanding of engagement. Third, there may be inherent selection bias introduced by social media advertising algorithms, which can influence which users are shown the advertisements based on prior behavior, platform usage patterns, or inferred interests. This may limit the generalizability of our findings to the broader target population.

In conclusion, we successfully disseminated two validated CPF educational videos and the accompanying Heal My Fistula website^7^ on Facebook and Instagram. These resources can help individuals with CPF better understand their condition and treatment options, empowering them in their own care. Our study also provided valuable insights into audience behaviors and preferences when engaging with online CPF content. Broad, general overview materials seemed to generate more engagement than treatment-focused content. Additionally, we found that older age groups and females exhibited the highest levels of engagement across both videos, suggesting that online content development should consider oversampling these demographics to better address their needs.

## Funding

This study was funded by a grant from by Takeda Pharmaceuticals, U.S.A., Inc. and was not a sponsored study.

## Data Availability

Data supporting the findings of this study are available from the corresponding author upon reasonable request.
